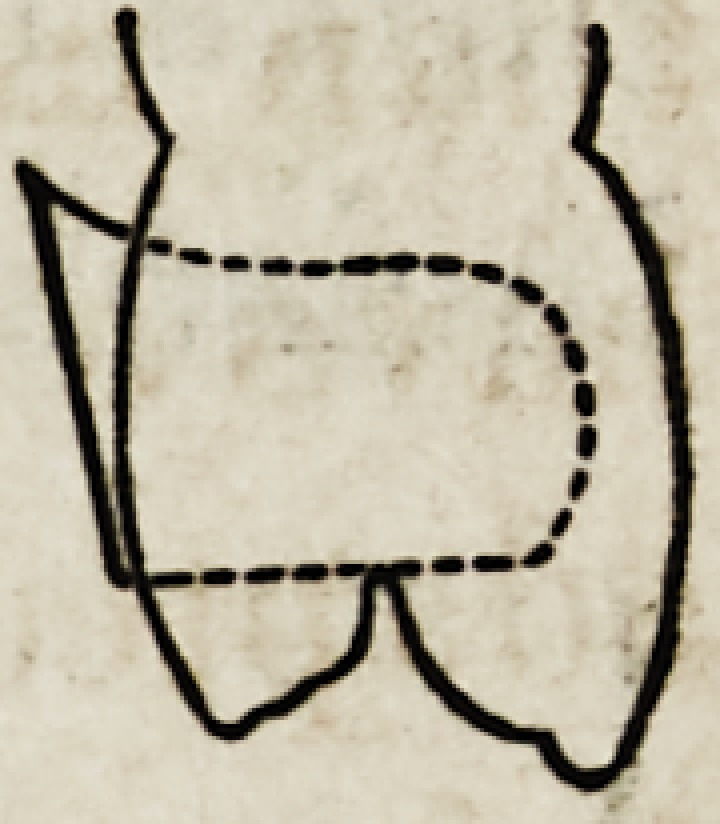# "Dentistry as a Fine Art." No. 3

**Published:** 1867-08

**Authors:** Norman W. Kingsley

**Affiliations:** Professor of Dental Art and Mechanism, in the New York College of Dentistry.


### AMERICAN JOURNAL

OF

### DENTAL SCIENCE.

Vol. I. THIRD SERIES.—AUGUST, 1867. No. 4.

### ORIGINAL COMMUNICATIONS.

### ARTICLE I.

### "*Dentistry as a Fine Art.*" *No.* 3.

By NORMAN W. KINGSLEY, Professor of Dental Art and Mechanism, in the New York College of Dentistry.

IN the preceding articles we have endeavored to make the distinction between dental "Art" and dental "Mechanism" clear and unmistakable, claiming for dental art a wider range than is generally accorded to it.

The fundamental principles which govern it being the same as those which govern other departments of Fine Arts, we quoted language which had been applied to them.

Principles, are necessarily general in their character.

Their special relation to our own department, we propose to consider in the following pages.

In the construction of an artificial denture, everything that relates to its appearance, belongs to Art; everything that affects its utility, is controlled by mechanism.

It is not only possible therefore, but very common to see, artificial teeth that are worn with great comfort and may be as serviceable as any that can be made, and not a single element of true art has entered into their construction.

The adaptation to the jaws, and the articulation for masticating purposes, in these days of plastic materials, involves, no skill beyond that possessed by many a carpenter throughout the land ; but the form and color of the teeth selected, their arrangement with each other, and the adaptation of the whole to the demands of the unimpaired features, present an appearance which is a grim satire upon Dentistry as an Art.

With the methods of construction now in common use, and with the expectations of patients, in regard to time, and remuneration, it is a difficult task to remedy the evil.

But the fault lies with the patient only to a limited extent ; with the ordinary facilities given to the dentist, he fails most signally to make the most of them. The taste of the profession is at a low standard. The teeth often selected from a large variety, show such a want of an appreciation of the fitness of things, that it is a wonder that they are acceptable to the patient.

It may be argued that, with the present practice of depending upon manufacturers for a supply, it is impossible to make from a limited stock just what the case in hand demands, and thus the dentist would throw upon the manufacturer the responsibility of the failure. It is very true that the stock of artificial teeth, of any or all the manufacturerers has never come up as a whole to what ought to be the requirements of an educated and appreciative profession, but it is after all not so much the fault with the individual productions in the market, as it is the total want of taste, and artistic skill in the adaptation of even imperfect means. There has not been a time within fifteen years but what teeth have been made which could be so arranged as to fulfil to a considerable extent, an artist's ideal.

While the productions of the manufacturer have been, and are still, exceedingly faulty as perfect imitations of nature ; they have been, we have reason to believe, fully up to the requirements of a profession devoid of æsthetic culture.

Taking the profession as a whole, the manufacturers have probably in this respect been the educators, rather than the followers.

This is evidently reversing the natural order of things. Manufacturers are but commercial men actuated by the love of gain, governed by the laws of trade—demand and supply—and it is a shameful comment upon a profession of the pretensions of Dentistry that a trade which cares only to supply what is demanded, should have the credit of teaching it its own wants. What shall be said of the judgment and taste that dictates such orders as this to a Dental Depot?

"Please send me a set of teeth for a clergyman's wife of medium size, not too dark." (No sample accompanying the order), or this—"Send me by Express C. O. D. a set of teeth for a young lady with blue eyes." Further than this, what will be thought of the artistic excellence of the productions of the dentist who, as is the case in one of our large cities with access to an almost unlimited stock, puts in by the aid of workmen seventy-five sets per week? Where in such cases is the "Fine Art" of Dentistry which requires the same study of the individual as is necessary to paint or model a portrait?

As this article is intended to deal only with *Art*, we will consider first, its simplest developements—that which is the least removed from mechanism, and proceed to those requirements which are more subtle and complicated.

The making of an artificial tooth is purely the performance of a sculptor. To produce the original model when the market is to be supplied with duplicates calls into exercise the same talents. To *copy* carefully however the various forms of teeth as they are presented, is Art, only in a very limited sense.

To carve an *imitation* of a natural denture—not a copy of any specific presentation, which shall in each individual tooth possess a character in harmony with the whole number, and with the face; to so arrange the whole as to assist in the very best expression of the surrounding features, and in addition, to give them the tone and color of nature ; is an artistic accomplishment in the highest sense.

Let us be fully understood as to the distinction we here make between *copy* and *imitation.*

"Servile copying and elaborate detail require no effort of skill beyond the attainment of the most limited capacity."

Copying is simply a mechanical achievement.

In all larger objects the perfection of the duplicate can be ascertained by measurement: machines are now made to duplicate almost any irregular form that is required. In smaller objects, a correct eye to detect variations takes the place of instruments.

A copy admits of no ideal embellishment.

In making a copy the mind is a slave ; but in creating an imitation the mind works with a freedom from all restraint. The true artist therefore rises above a mere copyist, and acquaints himself fully with nature in all her variety of developement.

He commits nature to memory in all her moods and diversities, and out of this storehouse, brings forth his *imitation*—which is in fact a new creation, and not the copy absolutely of anything. In the production of artificial teeth to supply the market but little art is required. The exercise of good judgement in the selection of natural organs to be duplicated in form and color, does not call into use the highest artistic talent. Artificial teeth when made by manufacturers should be in appearance, so far as they will be exposed in service, strictly copies from nature. We say *copies ;* because the manufacturer cannot by any possibility take cognizance of the peculiarities of the individual for whom they will be used. He cannot therefore indulge in an imitation, and benefit the dentist so much as by strictly duplicating nature, in a full variety, and leave to the dentist to hide as far as possible the individual incongruities by an artistic arrangement. It is somewhat surprising with such facilities for accomplishing this result, so far as forms are concerned, that the teeth in the market are so far short of the true standard.

Take for example the teeth without gums; the shape of the four incisors is generally very fair, but of the remaining number a large proportion bear but little resemblance to the organs they were intended to substitute.

The canines, which have individually more character than the others, and which have also more influence over the expression of the mouth, are in these productions as devoid of these qualities as a white bean. Of the bicuspids also, it is often very difficult to decide for which side of the mouth they were intended, and in some cases where the colour is uniform they might be inverted in the setting without any detriment to their appearance. While the manufacturers have undoubtedly done as much for the improvement of their productions as the mass of the profession would appreciate, there is still a great deal in this direction that remains to be done.

In the manufacture of teeth without gums either for plate or plastic work, there is no serious difficulty in carrying them almost to perfection. A faithful copy of a well selected variety of natural organs would accomplish the result.

The writer takes pleasure in bearing testimony to the great advance made in this direction a few years since by Dr. John M. Crowell, at that time engaged in the manufacture of teeth, who presented to the profession, forms of teeth superior to any before introduced. These forms were not only artistic improvements, but they bore unmistakable evidence of having been modeled or copied from nature. The peculiarities which they presented have been imitated by other makers, sometimes to such an extent as to be simply a caricature of the natural organs.

As has been before intimated, manufacturers make what will sell, and it is not to be wondered at that they should continue to fill the market with inferior productions so long as they find sale for them. But it is to be wondered at that a profession that is brought into daily contact with the natural teeth, and should be distinguished for its good taste, are such partial observers as not to detect the inferiority.

This lack of cultivation is evidenced in other ways besides the one referred to. In a majority of the publications where engravings of the teeth are used as illustrations—the forms are positively ugly; and it is not the fault of the engraver—he follows copy closely ; even to the imperfections. In the illustrations of the correction of irregular dentures, the models furnished the engraver, while conveying some notion of the change which has occurred, show in a majority of instances a disregard of the form of the teeth which would otherwise make the illustration much more effective. It is easy to see in many cases that the impression from which the model was made, was taken in wax and all the defects made by the draft of the wax in the removal, are shown in the model ; left untouched, and carefully copied by the engraver. This lack of appreciation of the beautiful, graceful and true, lies clearly with him who furnishes the model.

The beneficial influences upon the mind of having it fully impressed with an ideal standard, are not inconsiderable.

It becomes a great help in the determination of any type to be used or adapted to any given case.

With the mind thoroughly conversant with any given standard of excellence, it becomes very easy by the laws of the association of ideas, to make, or select teeth with such deviations from it as may be desirable. It will be remembered that the most pleasing forms in nature are those with the softest and most graceful outlines—hard and angular forms do not give pleasure except by contrast.

In the developement of the natural teeth the laws of harmony as universal in uninterrupted nature are beautifully illustrated. In the youth from twelve years old and upward, the features of the face present their most charming appearance: all the lines are soft and rounded; sharpness and angularity come on with maturity and old age.

The teeth obey the same law. In youth, immediately after their full eruption, they present their most perfect appearance; their cutting edges and grinding surfaces are beautifully modelled; but as age advances the abrasion of the antagonizing teeth, together with the almost imperceptible friction of one against another in the same row, continually act so as to modify this form.

Thus, in taking the extremes we find the perfection of full developement in the youth, changed to a mere stump without beauty in old age.

To describe all the types that are found in nature and which may be in perfect harmony with the surrounding features would be impossible. It would be assumption to give any one as possessing all excellence, but as in art there may be a standard or ideal, accepted by a majority of cultivated people, so we may present a type which shall combine the beauties of many, and from which deviations may be made as circumstances require.

Figures 1, 2, 3, 4 and 5, show the front view of two canines, a central and lateral incisor, and a bicuspid.

They are drawn larger than nature to render their peculiarities more forcible.

1 2 3 4 5 6 7 8

It will be seen that neither in their outlines nor any portion of their surface, are there straight lines or angles; every portion of the surface presents that easy and graceful contour, an artist loves to dwell upon.

The outlines of the incisors which are less undulating than those of any other are still far from square or angular.

Each side is unlike any other side, and the cutting edge which becomes square from abrasion as age advances, is, when fully developed, curved and wavy; and this line, fuller in the centre and depressed each side, is continued up the face of the tooth forming a gentle ridge perpendicularly along its surface.

The narrower and rounder parts of the tooth, will also be observed; the changes from the flatter portions coming not by regular inclination but at a point about two-thirds the length of the crown from the cutting edge, the outline dips by a graceful sweep into a depression, which is common to all well formed teeth.

This line of beauty is very often neglected in artificial teeth, when arranged in a denture with the shape as given by the mould the spaces between them have the appearance of being made with a separating file; so perfectly uniform are they.

All the teeth anterior to the molars have a ridge more or less perceptible running perpendicularly along the face of the tooth—this is sometimes very faint in the incisors, but is shown very bold and in striking contrast in the canines. In the incisors and bicuspids it always assumes a curve with an inclination toward the median line; but with the canines, this order is reversed, and the ridge curves the other way: thus

The central and lateral incisors, as any ordidary observer will have noticed, are very much the same in their general contour: the principle difference being, that the laterals are not quite as wide in proportion to their length and are about one-third narrower than the centrals.

In figures 2 and 5 are represented two types of canines. Figure 2 harmonizes better with the incisor shown here, than does figure 5.

Figure 5, would be more appropriately classed with longer and slimmer associates.

The characteristics of canine teeth are equally developed in both. The same graceful lines of beauty that marked the incisors are here also seen—the same depression on the sides of the upper third: the chief difference being that the canines at that point are rounder and bolder than the incisors; but below the upper third the difference is radical. The central ridge is very prominent and terminates in a cusp and the wavy line of the cutting edge of the incisors is duplicated, one on each side of the cusp, thus.

The posterior approximal surface is distinguished by a symetrically formed tubercle, more or less defined: but most certainly a mark of beauty. This tubercle is better delineated in figure 7 which is a profile view of figure 2. In figure 5, this tubercle with its corresponding prominence on the anterior approximal surface, is developed higher up on the tooth; which constitutes the main difference in the two types. In figures 1 and 8, we have a pure type of a bicuspid; the resemblance to the canine being easily seen: the same bold surface, cusp, undulated outline and posterior tubercle: the chief deviation in the external appearance being in a pretty well defined tubercle on the anterior approximal surface; and a relative reduction in size.

The characteristics of these three classes of teeth viz: incisors, canines and bicuspids are not confined to their front view.

Their profiles are equally peculiar; as shown in figures 6, 7 and 8. The central face of the incisor shows a regular curve; the canine has no less than three different planes or curves; the boundary between the upper third, and that below being marked by a decided prominence, while at the corresponding point on the bicuspid the profile is flat and the main fullness is below. The peculiarities thus pointed out, are all that concern the appearance of artificial teeth.

The second bicuspid does not differ materially from the first, and the molars are placed so far back as not to call for any especial criticism upon their appearance.

In passing we desire to call attention to a point that is almost always overlooked by the mere mechanical dentist.

The profile of the lingual surface is almost invariably *curved*, very *rarely* straight.

These teeth are oftener used to pass a clasp around than any other, and in a majority of instances the clasp at that point is made *flat;* and of course fits the teeth very inadequately,  or thus. 

The trouble arises from a supposition that the model is perfect, whereas if the impression is taken in wax, the model is sure to be faulty, and it is very often the case even with plaster impressions; and again, a lack of observation as to the real form, so that the model may be trimmed if defective.

The writer has hesitated, as to whether this article will permit of any suggestions or criticism, upon the color and tone, of artificial teeth, which will be of any benefit to the student.

That it is, in many respects, of equal or more importance than individual form, is undoubted, for with an artificial denture, faulty in form and weak in arrangement; if the color and tone exhibit good taste in the selection, it is a redeeming trait and worthy of praise. But as the faintest shades are of so much importance in this matter, and as they are so undefinable; the names of colors and their variations often conveying a different idea from what was intended that it is impossible to give more than the general suggestions of good taste. The manufacturer as well as the dentist, must in this especially, be a close student of nature, for only by long observation can the eye be cultivated to the nice discrimination essential to success.

One of the greatest difficulties to overcome is the scientific one, viz: to discover and combine in just proportions the materials that shall produce this wonderful imitation.

In no other art with which the writer is acquainted, has imitations of nature, been carried even now to such perfection. The making of artificial flowers has, perhaps, come the nearest to it. Certain it is, that of the materials which chemistry has already furnished us, it is possible to obtain most wonderful results.

For some cases the teeth made by Mr. Ash of London seem to be a closer imitation in their bony appearances, than most of our own productions ; out of the mouth they have a peculiarly vital appearance, but they are so illy adapted to the methods of making dentures common in this country that they have not to any extent come into use. The color of a tooth is dependent principally upon the proportion of its ingredients ; its tone upon the action of the fire in burning or baking.

The fault of many of the porcelain teeth of this country, is a crudeness or rawness in their appearance—a lack of translucency, which a little more heat would very much improve. It would blend the colors more perfectly, give them more vitality, and soften down the hard and angular lines of the the mould. It is perfectly in the power of our manufacturers with the materials now in use to make a general improvement.

One thing which is much wanted, is to increase the variety of darker shades ; not by hurrying into the market a lot of poorly baked, blue or yellow teeth ; but by a careful imitation of those organs in persons who have been habitually neglectful, until their teeth have acquired a tone or color which cannot be removed. While the dentist at large is dependent upon the manufacturer, he must cultivate his taste until he is able to select the most suitable shade which is prepared for him. When one or more of the front teeth are remaining either above or below, in a fair state of preservation, a tolerably correct idea may be gathered of what is needed ; and careful observation made of just such cases, as well as of all partial sets, taking into consideration, the age, complexion, &c., will do much to improve his judgment and enable him to make suitable adaptations when he has no such help. A few suggestions will be manifest to almost every one. Fair teeth are admissable in younger persons ; deeper hues are required for the aged. While we sometimes find in old persons, natural teeth, very fair to look upon, there is a seeming incongruity about it which we are not justified in imitating. It is safer to err upon the side of inserting those of a deeper tone than is really required. Excepting when some of the natural teeth remain, and then faithfully match or at least select a color that harmonizes, and will not be obtrusive or conspicuous. In all works of art, the subdued tones are the most permanently pleasing.

(To be Continued.)

## Figures and Tables

**1 f1:**
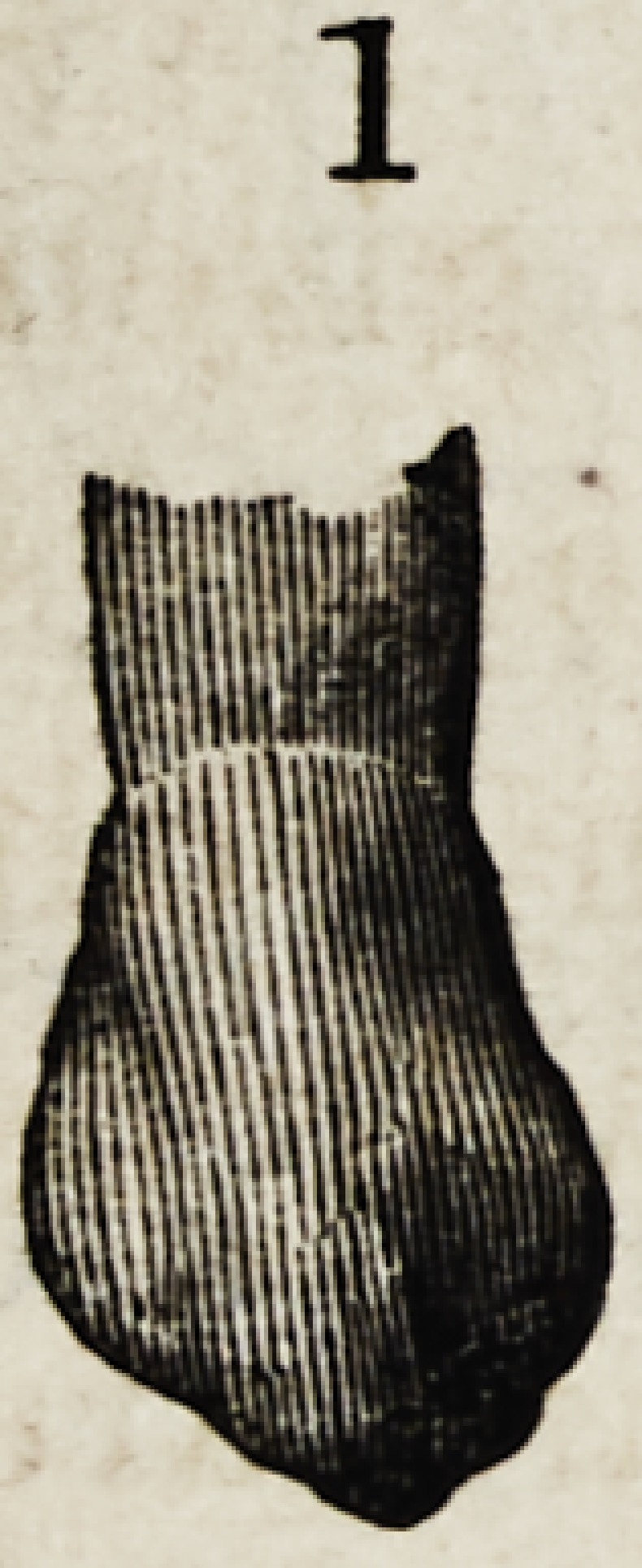


**2 f2:**
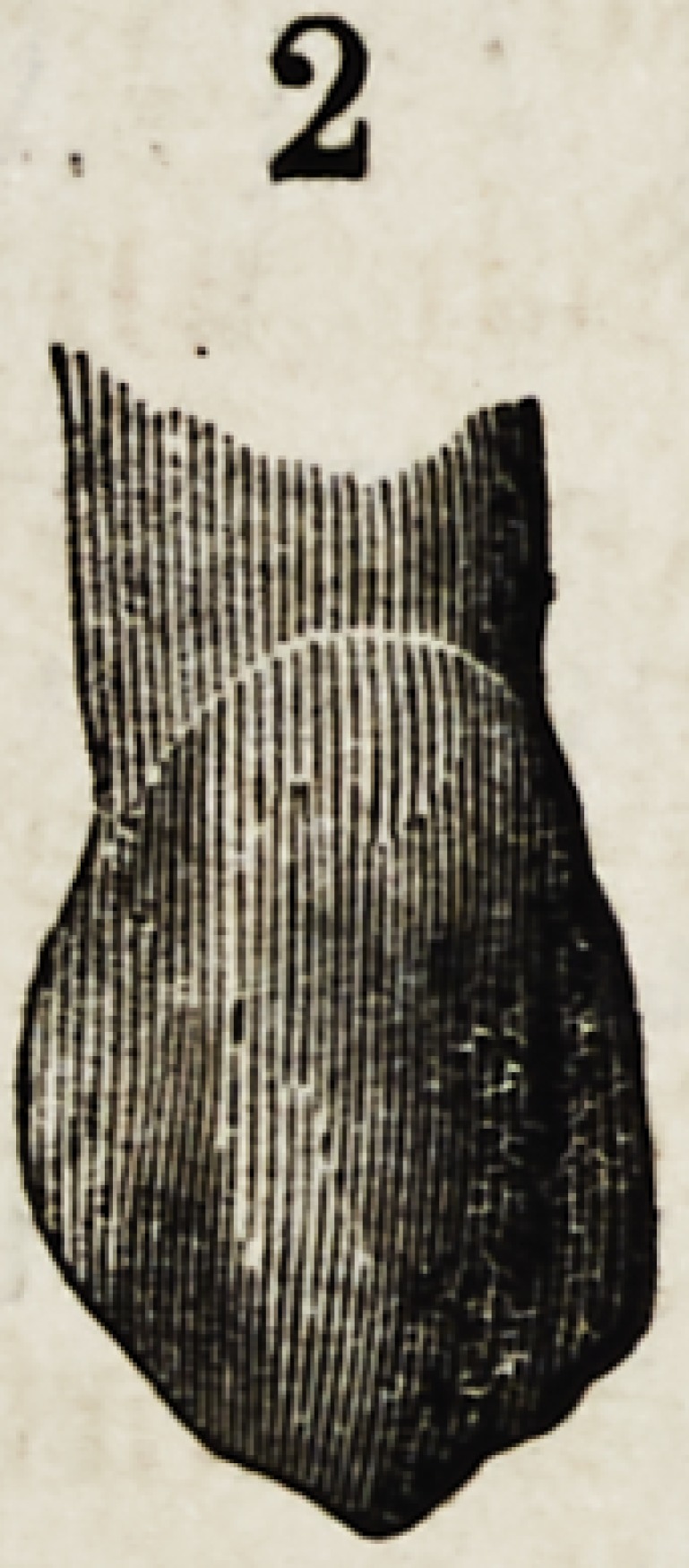


**3 f3:**
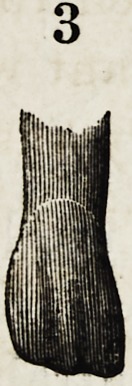


**4 f4:**
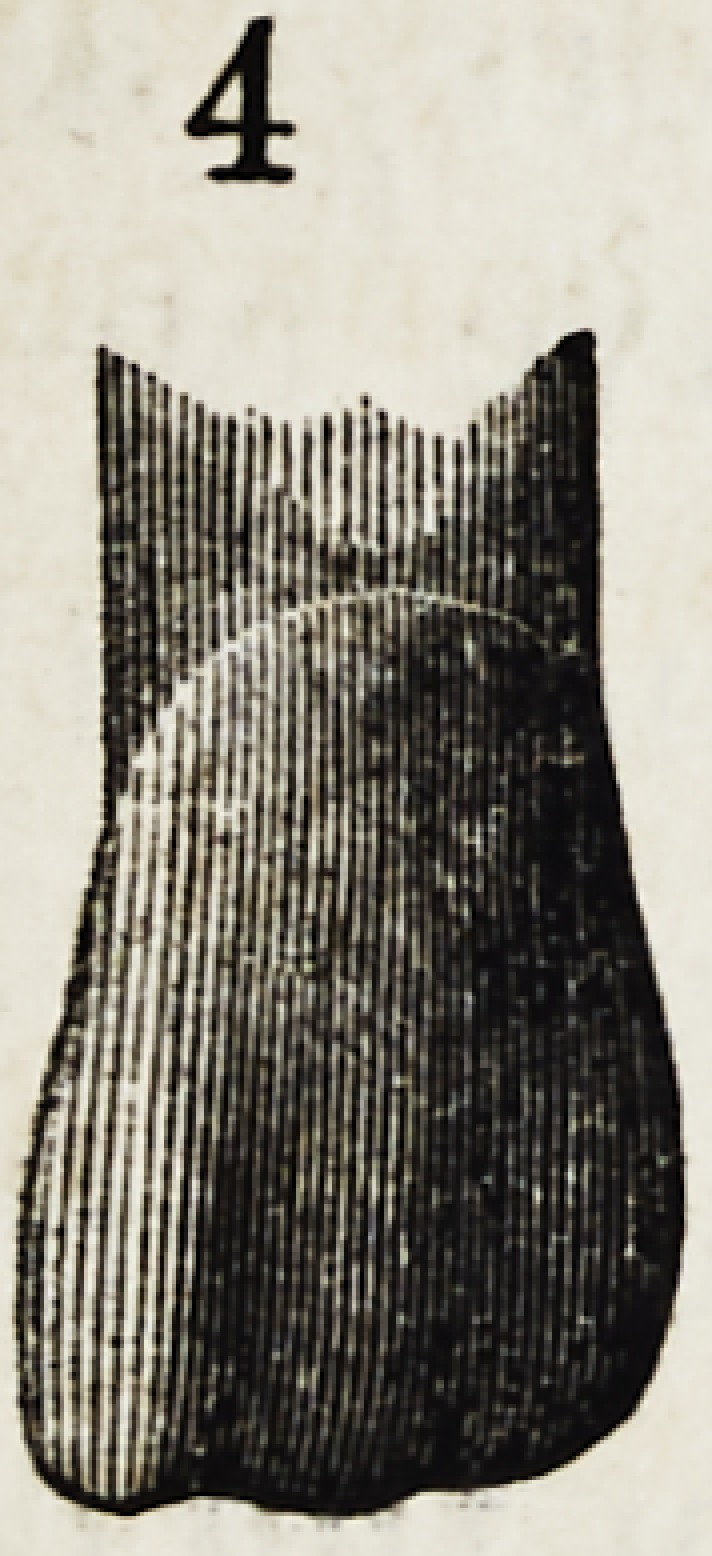


**5 f5:**
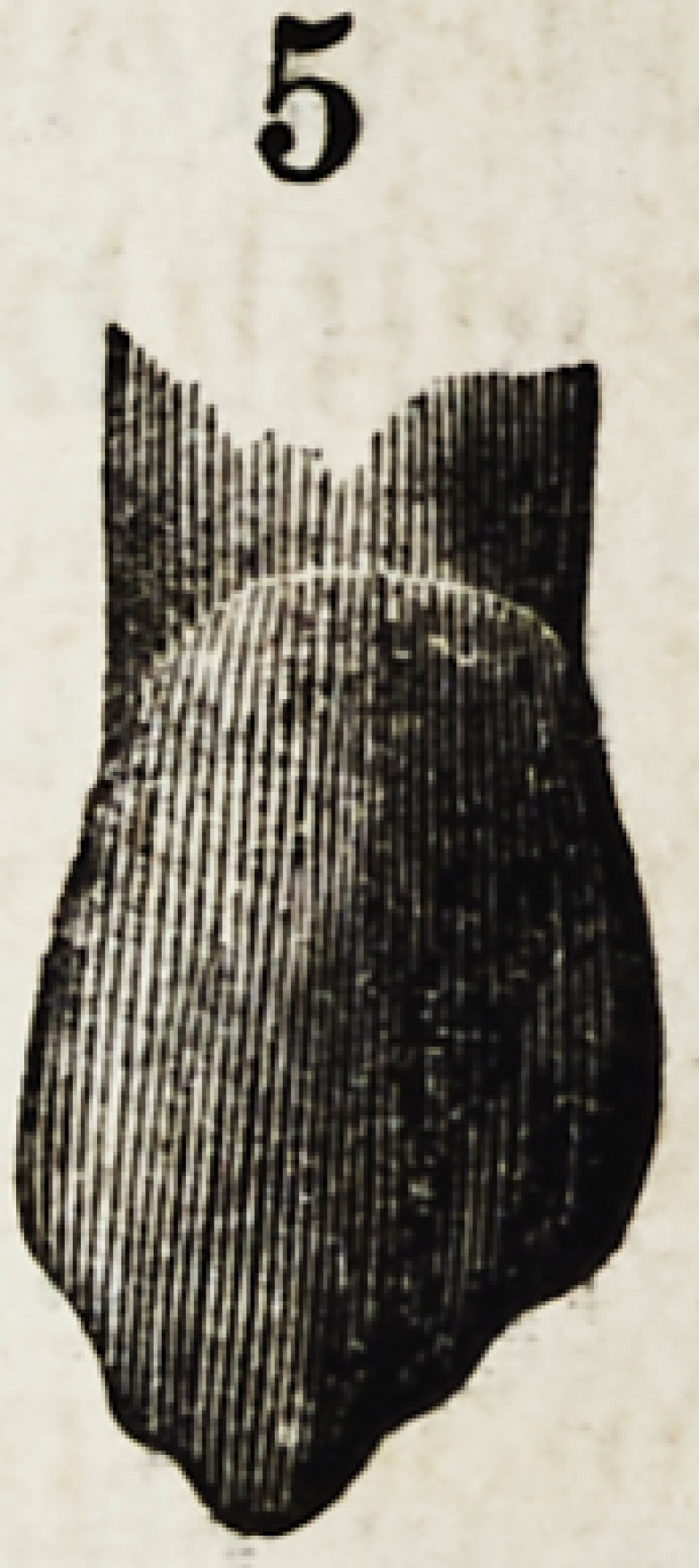


**6 f6:**
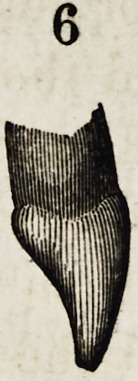


**7 f7:**
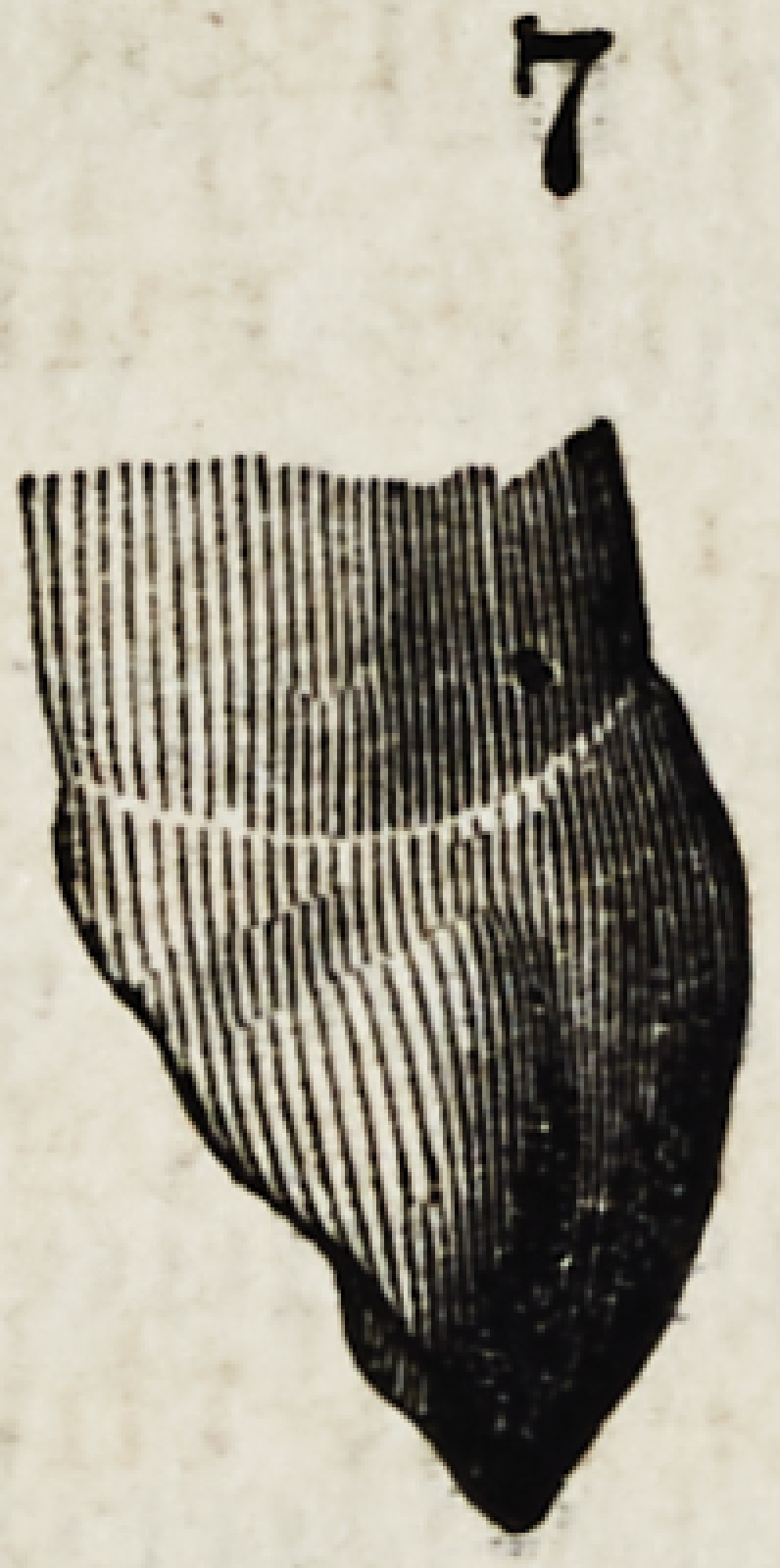


**8 f8:**
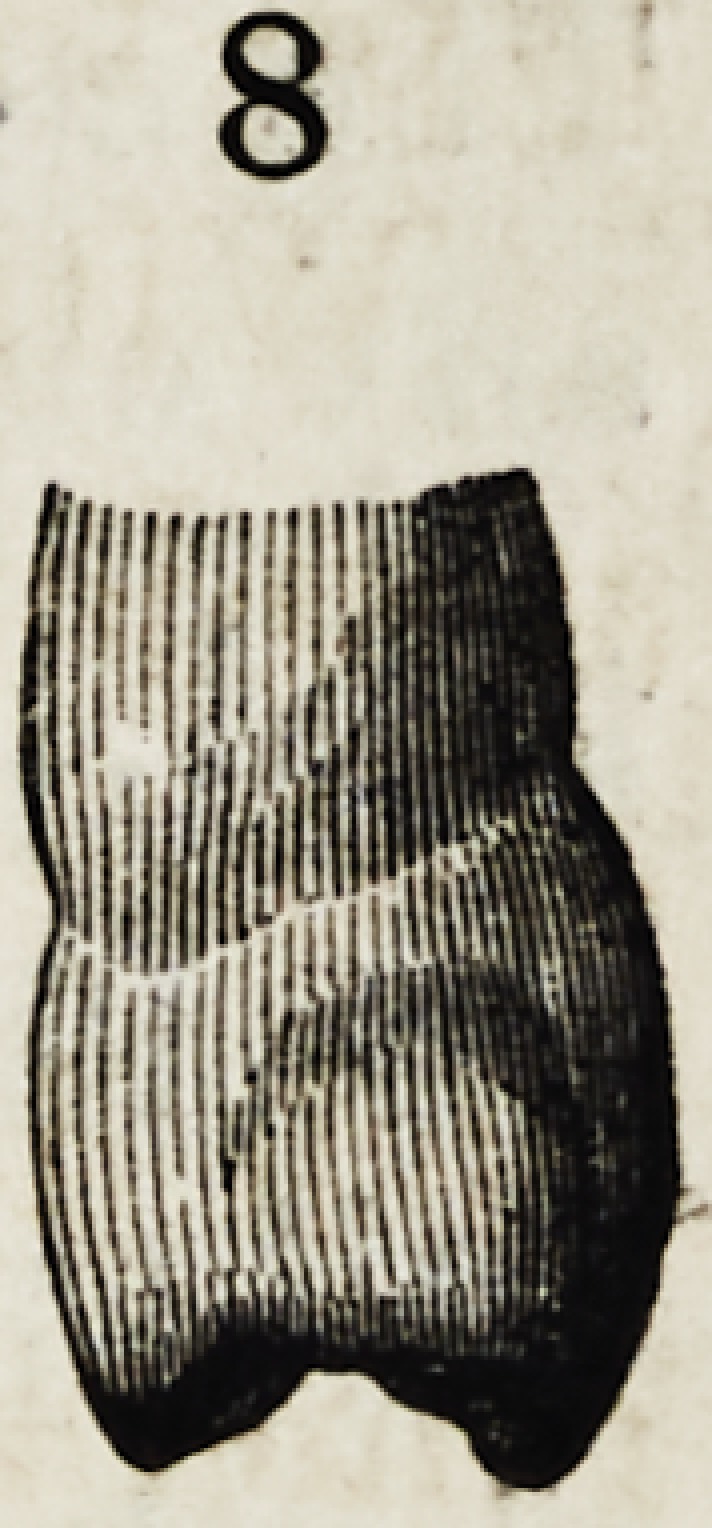


**Figure f9:**
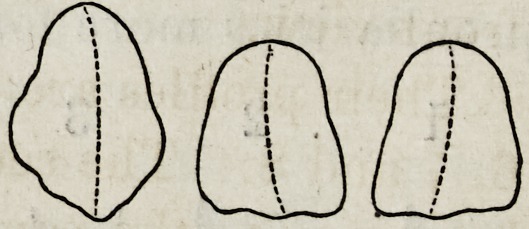


**Figure f10:**
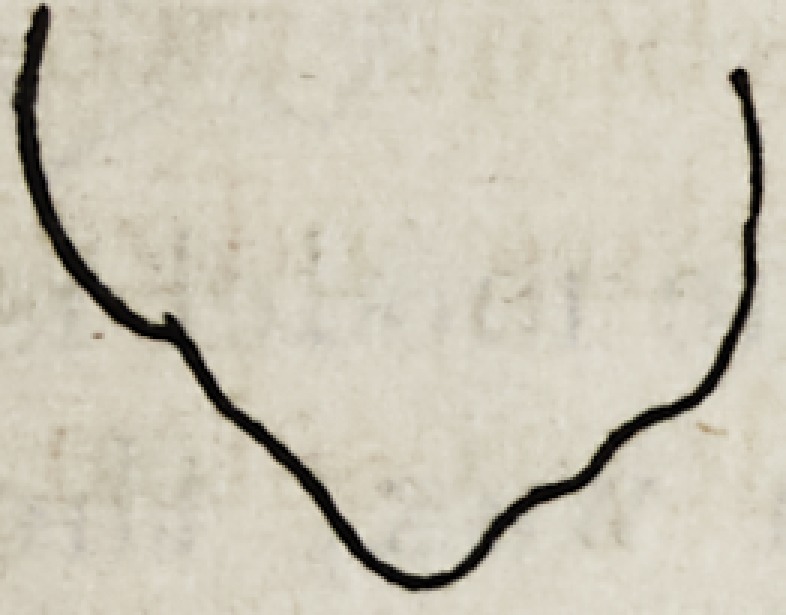


**Figure f11:**
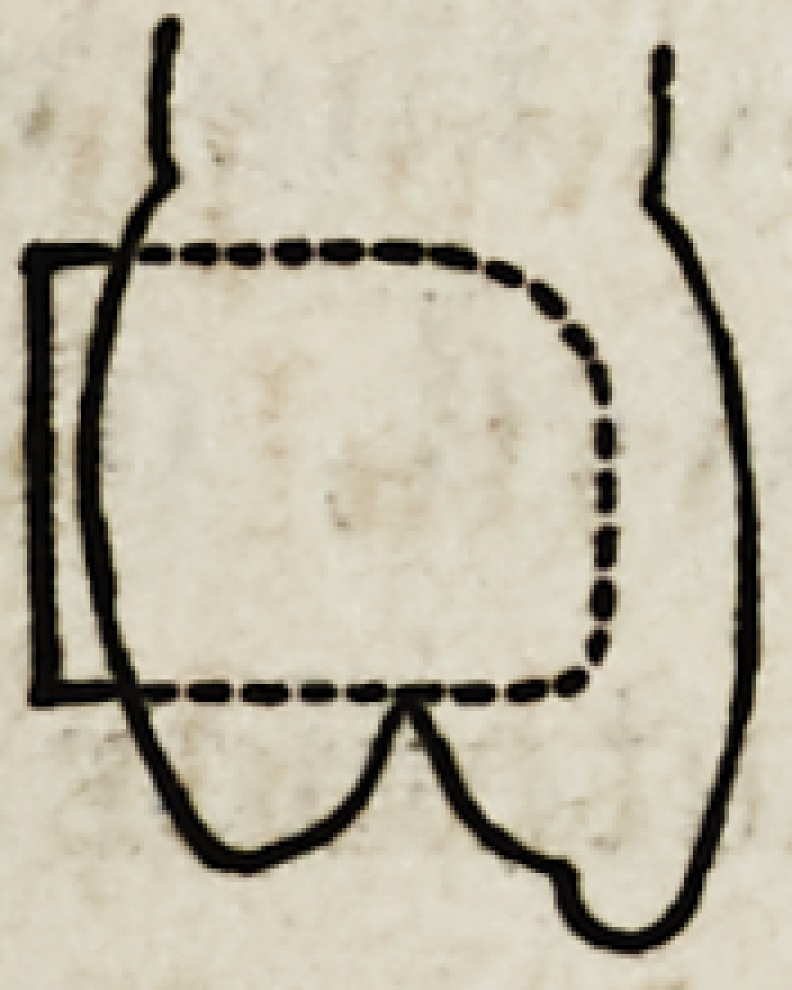


**Figure f12:**